# Supplementary material for: Hedges, mottes, and baileys: Causally ambiguous statistical language can increase perceived study quality and policy relevance
Source: PLoS One. 2023 Oct 26;18(10):e0286403. doi: 10.1371/journal.pone.0286403 (PMC10602341; doi:10.1371/journal.pone.0286403)
Supplement: S1 File — (DOCX) [file pone.0286403.s001.docx]

Online Supplementary Materials

**Table S1**

*Descriptive Statistics for the Demographic Variables of the Preregistered Sample*

| Variable | | *n* | % | *M (SD)* |
| --- | --- | --- | --- | --- |
| Condition | A | 18 | 33 |  |
|  | B | 17 | 27 |  |
|  | C | 11 | 18 |  |
|  | D | 12 | 22 |  |
| Source | cogdevsoc listserv | 29 | 47 |  |
|  | PsychMD | 16 | 29 |  |
|  | Twitter | 13 | 24 |  |
| Sex | Male | 22 | 38 |  |
|  | Female | 35 | 60 |  |
|  | Non-binary | 1 | 2 |  |
| Age |  | 56 |  | 33.77 (9.67) |
| Statistical Courses Taken |  | 58 |  | 4.78 (3.17) |
| Country Academic Career | United States | 40 | 67 |  |
|  | Canada | 6 | 11 |  |
|  | Other / Did not answer | 9 | 22 |  |
| Age Learned English | Native Speaker | 37 | 64 |  |
|  | Before Age 6 | 6 | 10 |  |
|  | Between Ages 7-10 | 9 | 16 |  |
|  | Between Ages 11-14 | 6 | 11 |  |
|  | Between Ages 15-18 | 0 | 0 |  |
|  | Prefer not to answer / Did not answer | 0 | 0 |  |
| Career Status | Faculty | 20 | 35 |  |
|  | Postdoctoral Researcher | 15 | 27 |  |
|  | PhD Student | 21 | 38 |  |
|  | Masters Student | 1 | 0 |  |
|  | Undergraduate | 0 | 0 |  |
|  | Other / Did not answer | 2 | 0 |  |
| Academic Field | Psychology | 58 | 85 |  |
|  | Other / Did not answer | 0 | 0 |  |
| Psychology Subfield | Clinical | 2 | 2 |  |
|  | Cognitive | 15 | 25 |  |
|  | Neuroscience | 3 | 4 |  |
|  | Developmental | 29 | 47 |  |
|  | Quantitative | 6 | 11 |  |
|  | Social/Personality | 8 | 15 |  |
|  | Educational | 5 | 9 |  |
|  | Other / Did not answer | 6 | 0 |  |
| Passed Manipulation Check |  | 58 | 41 |  |
| Participants excluded |  | 84 | 57 |  |
| Note. *n* = 58. PsychMD = Psychological Methods Discussion Facebook group. Descriptive statistics of full pre-registered analytic sample after cases that met exclusionary criteria were removed. Participants that preferred not to answer questions or did not answer were reported together to prevent any possibility of identification. Some academic fields categorized as other included 6 areas that were included in the analyses as psychological subfields. | | | | |

**Table S2**

*Descriptive Statistics for Survey Responses from the Pre-registered Analytic Sample Meeting Inclusionary Criteria*

| Variable | *n* | *M (SD)* | *n* | *M (SD)* |
| --- | --- | --- | --- | --- |
| Abstract 1 : Scientific Reasoning |  |  |  |  |
| Statistically Ambiguous Language | 29 | 3.31 (0.66) | 29 | 2.55 (0.91) |
| Causal Language | 29 | 2.72 (0.65) | 29 | 1.93 (0.80) |
| Abstract 2 : Reading Achievement |  |  |  |  |
| Statistically Ambiguous Language | 29 | 3.41 (0.57) | 29 | 2.34 (1.04) |
| Causal Language | 29 | 2.93 (0.65) | 29 | 2.38 (1.05) |
| Overall |  |  |  |  |
| Statistically Ambiguous Language | 58 | 3.36 (0.61) | 58 | 2.45 (0.98) |
| Causal Language | 58 | 2.83 (0.65) | 58 | 2.16 (0.95) |
| Note. *n* = 58. Descriptive statistics of full pre-registered analytic sample after cases that met exclusionary criteria were removed. 87 cases were excluded because participants answered first two questions in under 30 seconds (n=12), participants were not in a field related to psychology (n=3), participants were not doctoral students, post-doctoral researchers, or faculty (n = 6), or they failed the manipulation check (n=79); there was overlap across the participants reasons for exclusion. Question 1:“Based on the limited information available in this abstract, how would you rate the quality of the design and analysis of the study described?” is answered on a scale from 1 – 5 where 5 =Very high quality, 4 = High quality , 3 = Moderate quality , 2 = Low quality, and 1= Very low quality . Question 2: “Based on the limited information available in this abstract, how strongly does the study support the conclusion in the final sentence, quoted below: “We conclude that raising the quantity and quality of (resources for third grade reading OR early science instruction) is an important way to raise children’s educational prospects in the 21st century.” Is answered on a scale from 5 = Very strongly, 4 = Strongly, 3 = Moderately, 2= Weakly, 1 = Very Weakly/Not at all. | | | | |

**Table S3**

*Ordered Logit Models of Participant Ratings of Quality of Design and Study*

| **Model** | **1** | | | | **2** | | | | **3** | | | | **4** | | | |
| --- | --- | --- | --- | --- | --- | --- | --- | --- | --- | --- | --- | --- | --- | --- | --- | --- |
|  | **Coeff** | **SE** | **OR** | **CI** | **Coeff** | **SE** | **OR** | **CI** | **Coeff** | **SE** | **OR** | **CI** | **Coeff** | **SE** | **OR** | **CI** |
| Causally Ambiguous Statistical Language | 1.06*** | 0.25 | 2.89 | [1.78,4.67] | 1.17** | 0.35 | 3.22 | [1.62,6.43] | 0.93** | 0.31 | 2.55 | [1.40,4.63] | 1.82*** | 0.45 | 6.19 | [2.59, 14.81] |
| Abstract | -0.011 | 0.24 | 0.98 | [0.62,1.57] | -0.02 | 0.33 | 0.97 | [0.51,1.88] | 0.25 | 0.29 | 1.29 | [0.72, 2.30] | 0.5 | 0.39 | 1.64 | [0.76, 3.54] |
| Order | 0.48* | 0.24 | 1.62 | [1.02, 2.58] | – |  |  |  | 0.5 | 0.3 | 1.65 | [0.92, 2.96] | 0.66 | 0.4 | 1.94 | [0.89, 4.20] |
| Degrees of Freedom | 132 |  |  |  | 132 |  |  |  | 90 |  |  |  | 55 |  |  |  |
| Observations | 260 |  |  |  |  |  |  |  |  |  |  |  |  |  |  |  |
| Model Specifications | | | | | | | | | | | | | | | | |
| Both abstracts | X | | | |  | | | |  | | | |  | | | |
| First abstract only |  | | | | X | | | |  | | | | X | | | |
| Passed Manipulation check |  | | | |  | | | |  | | | | X | | | |
| First 100 participants |  | | | |  | | | | X | | | |  | | | |
| Passed exclusionary criteria |  | | | |  | | | |  | | | | X | | | |

Note. *** p< 0.001, **p<0.01, *p< 0.05. Coeff = Ordered logit coefficient, SE= standard error, OR= odd’s ratio, CI = 95% confidence interval. Question 1:“Based on the limited information available in this abstract, how would you rate the quality of the design and analysis of the study described? “is answered on a scale from 1 – 5 where 5 =Very high quality, 4 = High quality, 3 = Moderate quality, 2 = Low quality, and 1= Very low quality. Causally Ambiguous Statistical Language is a dummy variable in which causal language used in the abstract = 0 and Causally Ambiguous Statistical Language = 1. The abstract variable is coded as 1 = abstract 1 and 2 = abstract 2 to indicate effects of abstract used on quality rating. Order indicates which abstract was seen first (1) or second (2) to measure the effects of abstract order on quality rating. Model 4 represents the preregistered sample, 84 cases were excluded because participants answered first two questions in under 30 seconds (n=12), participants were not in a field related to psychology (n=6), they failed the manipulation check (n=64), were not enrolled in a PhD program, post-doctoral position, or a faculty position(n = 1), or they were not at least 18 years-old (n=1). Our full sample size was 142, however 8 cases were missing the second abstract and were excluded from the analyses.

**Table S4**

*Ordered Logit Models of Participant Ratings of Perceived Support for Policy Conclusion*

| **Model** | **1** | | | | **2** | | | | **3** | | | | **4** | | | |
| --- | --- | --- | --- | --- | --- | --- | --- | --- | --- | --- | --- | --- | --- | --- | --- | --- |
|  | **Estimate** | **SE** | **OR** | **CI** | **Estimate** | **SE** | **OR** | **CI** | **Estimate** | **SE** | **OR** | **CI** | **Estimate** | **SE** | **OR** | **CI** |
| Causally Ambiguous Statistical Language | 0.36 | 0.23 | 1.43 | [0.92,2.25] | 0.4 | 0.32 | 1.49 | [0.79,2.80] | 0.33 | 0.28 | 1.39 | [0.81, 2.40] | 0.55 | 0.36 | 1.73 | [0.85, 3.48] |
| Abstract | 0.04 | 0.23 | 1.04 | [0.66,1.62] | -0.19 | 0.32 | 0.83 | [0.44, 1.55] | 0.16 | 0.28 | 1.17 | [0.68, 2.01] | -0.01 | 0.36 | 0.99 | [0.49, 2.00] |
| Order | 0.81*** | 0.23 | 2.25 | [1.43,3.54 | – |  |  |  | 0.73** | 0.28 | 2.08 | [1.21, 3.60] | 0.62 | 0.36 | 1.87 | [0.91,3.80] |
| Participants | 132 |  |  |  | 132 |  |  |  | 90 |  |  |  | 55 |  |  |  |
| Observations | 260 |  |  |  |  |  |  |  |  |  |  |  |  |  |  |  |
| Model Specifications | | | | | | | | | | | | | | | | |
| Both abstracts | X | | | |  | | | |  | | | |  | | | |
| First abstract only |  | | | | X | | | |  | | | | X | | | |
| Passed Manipulation check |  | | | |  | | | |  | | | | X | | | |
| First 100 participants |  | | | |  | | | | X | | | |  | | | |
| Passed exclusionary criteria |  | | | |  | | | |  | | | | X | | | |

Note. *** p< 0.001, **p<0.01, *p< 0.05. Coeff = Ordered logit coefficient, SE= standard error, OR= odd’s ratio, CI = 95% confidence interval. Question 1:“Based on the limited information available in this abstract, how would you rate the quality of the design and analysis of the study described? “is answered on a scale from 1 – 5 where 5 =Very high quality, 4 = High quality, 3 = Moderate quality, 2 = Low quality, and 1= Very low quality. Causally Ambiguous Statistical Language is a dummy variable in which causal language used in the abstract = 0 and Causally Ambiguous Statistical Language = 1. The abstract variable is coded as 1 = abstract 1 and 2 = abstract 2 to indicate effects of abstract used on quality rating. Order indicates which abstract was seen first (1) or second (2) to measure the effects of abstract order on quality rating. Model 4 represents the preregistered sample, 84 cases were excluded because participants answered first two questions in under 30 seconds (n=12), participants were not in a field related to psychology (n=6), they failed the manipulation check (n=64), were not enrolled in a PhD program, post-doctoral position, or a faculty position(n = 1), or they were not at least 18 years-old (n=1). Our full sample size was 142, however 8 cases were missing the second abstract and were excluded from the analyses.

**Table S5**

*Participant Ratings of Quality of Design and Study Split by Survey Media Source*

|  | Twitter | | |  | PsychMD | | |  | Cogdevsoc | | | |
| --- | --- | --- | --- | --- | --- | --- | --- | --- | --- | --- | --- | --- |
| Variable | Estimate | SE | Effect Size |  | Estimate | SE | Effect Size |  | Estimate | SE | Effect Size | |
| Causally Ambiguous Statistical Language | 0.22 | 0.15 | 0.31 |  | 0.58*** | 0.13 | 0.75 |  | 0.45** | 0.11 | 0.51 | |
| Abstract | 0.2 | 0.15 |  |  | 0.08 | 0.13 |  |  | -0.11 | 0.1 |  | |
| Order | 0.03 | 0.15 |  |  | 0.29* | 0.13 |  |  | 0.23* | 0.1 |  | |
| Intercept | 2.57*** | 0.25 |  |  | 2.51*** | 0.24 |  |  | 2.83*** | 0.19 |  | |
| Participants | 31 |  |  |  | 34 |  |  |  | 67 |  |  | |
| Observations | 61 |  |  |  | 65 |  |  |  | 134 |  |  | |
| *R^2^* | 0.05 |  |  |  | 0.18 |  |  |  | 0.09 |  |  | |
| **Model Specification** | | | | | | | | | | | |  |
| Both abstracts | X | | |  | X | | |  | X | | | |

*Note.* *** p< 0.001, **p<0.01, *p< 0.05. PsychMD = Psychological Methods Discussion Facebook group. The dependent variable Question 1: “Based on the limited information available in this abstract, how would you rate the quality of the design and analysis of the study described? “is answered on a scale from 1 – 5 where 5 =Very high quality, 4 = High quality, 3 = Moderate quality, 2 = Low quality, and 1= Very low quality Causally Ambiguous Statistical Language is a dummy variable in which causal language used in the abstract = 0 and Causally Ambiguous Statistical Language = 1. The abstract variable is coded as 1 = abstract 1 and 2 = abstract 2 to indicate effects of abstract used on support rating. Order indicates which abstract was seen first (1) or second (2) to measure the effects of abstract order on support rating. Model 1 is an ordinary least squares model using only the support response from the first abstract seen by the participants. All other models use individual random effects to estimate the effect of the independent variables on changes in support rating within-person since all participants saw to abstracts and provided two quality ratings. SD indicates one standard deviation for the outcome variable. An effect size can be calculated by dividing regression estimates by the outcome standard deviation. *R^2^* reflects the pseudo r-squared for fixed effects.

**Table S6**

*Participant Ratings of Perceived Support for Policy Conclusion Split by Survey Media Source*

|  | Twitter | | |  | PsychMD | | |  | Cogdevsoc | | | |
| --- | --- | --- | --- | --- | --- | --- | --- | --- | --- | --- | --- | --- |
| Variable | Estimate | SE | Effect Size |  | Estimate | SE | Effect Size |  | Estimate | SE | Effect Size |  |
| Causally Ambiguous Statistical Language | 0.04 | 0.16 | 0.04 |  | 0.05 | 0.17 | 0.05 |  | 0.32** | 0.11 | 0.33 |  |
| Abstract | 0.40* | 0.16 |  |  | -0.10 | 0.17 |  |  | -0.07 | 0.11 |  |  |
| Order | 0.54** | 0.16 |  |  | 0.27 | 0.17 |  |  | 0.55*** | 0.11 |  |  |
| Intercept | 1.24*** | 0.3 |  |  | 2.16*** | 0.32 |  |  | 1.86*** | 0.2 |  |  |
| Participants | 31 |  |  |  | 34 |  |  |  | 67 |  |  |  |
| Observations | 61 |  |  |  | 65 |  |  |  | 134 |  |  |  |
| *R^2^* | 0.12 |  |  |  | 0.02 |  |  |  | 0.11 |  |  |  |
| **Model Specifications** | | | | | | | | | | | | |
| Both abstracts | X | | |  | X | | |  | X | | | |

*Note.* *** p< 0.001, **p<0.01, *p< 0.05. PsychMD = Psychological Methods Discussion Facebook group. The dependent variable Question 2: *“Based on the limited information available in this abstract, how strongly does the study support the conclusion in the final sentence, quoted below: We conclude that raising the quantity and quality of early science instruction is an important way to raise children’s educational prospects in the 21^st^ century” OR “We conclude that extra resources for third grade reading instruction are likely to improve children’s college readiness, and propose this as a useful intervention*”, is answered on a scale from *5 = Very strongly*, 4 = *Strongly*, 3 = *Moderately,* 2= *Weakly,* 1 = *Very Weakly/Not at all*. Causally Ambiguous Statistical Language is a dummy variable in which causal language used in the abstract = 0 and Causally Ambiguous Statistical Language = 1. The abstract variable is coded as 1 = abstract 1 and 2 = abstract 2 to indicate effects of abstract used on support rating. Order indicates which abstract was seen first (1) or second (2) to measure the effects of abstract order on support rating. Model 1 is an ordinary least squares model using only the support response from the first abstract seen by the participants. All other models use individual random effects to estimate the effect of the independent variables on changes in support rating within-person since all participants saw to abstracts and provided two quality ratings. SD indicates one standard deviation for the outcome variable. An effect size can be calculated by dividing regression estimates by the outcome standard deviation. *R^2^* reflects the pseudo r-squared for fixed effects.

**Table S7**

*Percentage of Psychology Faculty, Post-doctoral scholars, and PhD students Split by Media Source and Psychology Subfield*

|  | cogdevsoc | | |  | PsychMD | | |  | Twitter | | |
| --- | --- | --- | --- | --- | --- | --- | --- | --- | --- | --- | --- |
| Psychology Subfield | Faculty | Post-Doctoral Researcher | PhD Student |  | Faculty | Post-Doctoral Researcher | PhD Student |  | Faculty | Post-Doctoral Researcher | PhD Student |
| Clinical | 4% | 0% | 14% |  | 14% | 0% | 0% |  | 0% | 0% | 33% |
| Cognitive | 35% | 25% | 38% |  | 0% | 29% | 33% |  | 38% | 50% | 25% |
| Neuroscience | 4% | 17% | 10% |  | 14% | 14% | 0% |  | 25% | 17% | 8% |
| Developmental | 87% | 92% | 81% |  | 0% | 0% | 50% |  | 50% | 50% | 42% |
| Quantitative | 4% | 0% | 5% |  | 57% | 14% | 33% |  | 13% | 0% | 8% |
| Social/Personality | 0% | 0% | 10% |  | 71% | 57% | 17% |  | 25% | 17% | 8% |
| Educational | 22% | 0% | 5% |  | 0% | 14% | 17% |  | 0% | 0% | 0% |
| Other | 0% | 7% | 5% |  | 11% | 13% | 17% |  | 0% | 0% | 0% |
| Missing | 15% | 7% | 0% |  | 11% | 0% | 25% |  | 10% | 0% | 8% |
| *N* | 27 | 14 | 22 |  | 9 | 8 | 12 |  | 10 | 6 | 13 |

*Note.* Full sample of 142 participants is shown. Participants self-reported professional status some participants opted to not answer the demographic questions. Participants were allowed to choose multiple subfields thus percentages do not always add up to 100%.

**Table S8**

*Participant Ratings of Quality of Design and Study Split by Participant Professional Status*

|  | Faculty | | |  | Post-Doctoral Researchers | | |  | PhD Students | | | |
| --- | --- | --- | --- | --- | --- | --- | --- | --- | --- | --- | --- | --- |
| Variable | Estimate | SE | Effect Size |  | Estimate | SE | Effect Size |  | Estimate | SE | Effect Size | |
| Causally Ambiguous Statistical Language | 0.49*** | 0.11 | 0.51 |  | 0.58** | 0.16 | 0.72 |  | 0.36** | 0.11 | 0.44 | |
| Abstract | -0.12 | 0.11 |  |  | 0.14 | 0.16 |  |  | 0.05 | 0.11 |  | |
| Order | 0.14 | 0.11 |  |  | 0.40* | 0.16 |  |  | 0.15 | 0.11 |  | |
| Intercept | 2.80*** | 0.13 |  |  | 2.46*** | 0.21 |  |  | 2.68*** | 0.13 |  | |
| Participants | 46 |  |  |  | 28 |  |  |  | 47 |  |  | |
| Observations | 92 |  |  |  | 56 |  |  |  | 94 |  |  | |
| *R^2^* | 0.11 |  |  |  | 0.15 |  |  |  | 0.07 |  |  | |
| **Model Specifications** | | | | | | | | | | | |  |
| Both abstracts | X | | |  | X | | |  | X | | | |

*Note.* *** p< 0.001, **p<0.01, *p< 0.05. The dependent variable is Question 1: “Based on the limited information available in this abstract, how would you rate the quality of the design and analysis of the study described? “is answered on a scale from 1 – 5 where 5 =Very high quality, 4 = High quality, 3 = Moderate quality, 2 = Low quality, and 1= Very low quality. Causally Ambiguous Statistical Language is a dummy variable in which causal language used in the abstract = 0 and Causally Ambiguous Statistical Language = 1. The abstract variable is coded as 1 = abstract 1 and 2 = abstract 2 to indicate effects of abstract used on support rating. Order indicates which abstract was seen first (1) or second (2) to measure the effects of abstract order on support rating. Model 1 is an ordinary least squares model using only the support response from the first abstract seen by the participants. All other models use individual random effects to estimate the effect of the independent variables on changes in support rating within-person since all participants saw to abstracts and provided two quality ratings. SD indicates one standard deviation for the outcome variable. An effect size can be calculated by dividing regression estimates by the outcome standard deviation (SD=.81). R2 reflects the pseudo r-squared for fixed effects.

**Table S9**

*Participant Ratings of Perceived Support for Policy Conclusion Split by Participant Professional Status*

|  | Faculty | | |  | Post-Doctoral Researchers | | |  | PhD Students | | |
| --- | --- | --- | --- | --- | --- | --- | --- | --- | --- | --- | --- |
| Variable | Estimate | SE | Effect Size |  | Estimate | SE | Effect Size |  | Estimate | SE | Effect Size |
| Causally Ambiguous Statistical Language | 0.24* | 0.12 | 0.30 |  | 0.18 | 0.19 | 0.22 |  | 0.21 | 0.13 | 0.26 |
| Abstract | -0.07 | 0.12 |  |  | -0.15 | 0.18 |  |  | 0.24 | 0.13 |  |
| Order | 0.32* | 0.12 |  |  | 0.66*** | 0.18 |  |  | 0.41*** | 0.13 |  |
| Intercept | 1.94*** | 0.16 |  |  | 1.66*** | 0.33 |  |  | 1.69*** | 0.15 |  |
| Participants | 46 |  |  |  | 28 |  |  |  | 47 |  |  |
| Observations | 92 |  |  |  | 56 |  |  |  | 94 |  |  |
| *R^2^* | 0.04 |  |  |  | 0.14 |  |  |  | 0.07 |  |  |
| **Model Specifications** | | | | | | | | | | | |
| Both abstracts | X | | |  | X | | |  | X | | |

*Note.* *** p< 0.001, **p<0.01, *p< 0.05. Effect size is standardized. The dependent variable Question 2: *“Based on the limited information available in this abstract, how strongly does the study support the conclusion in the final sentence, quoted below: We conclude that raising the quantity and quality of early science instruction is an important way to raise children’s educational prospects in the 21^st^ century” OR “We conclude that extra resources for third grade reading instruction are likely to improve children’s college readiness, and propose this as a useful intervention*”, is answered on a scale from *5 = Very strongly*, 4 = *Strongly*, 3 = *Moderately,* 2= *Weakly,* 1 = *Very Weakly/Not at all*. Causally Ambiguous Statistical Language is a dummy variable in which causal language used in the abstract = 0 and Causally Ambiguous Statistical Language = 1. The abstract variable is coded as 1 = abstract 1 and 2 = abstract 2 to indicate effects of abstract used on support rating. Order indicates which abstract was seen first (1) or second (2) to measure the effects of abstract order on support rating. Model 1 is an ordinary least squares model using only the support response from the first abstract seen by the participants. All other models use individual random effects to estimate the effect of the independent variables on changes in support rating within-person since all participants saw to abstracts and provided two quality ratings. SD indicates one standard deviation for the outcome variable for each analysis sample. An effect size can be calculated by dividing regression estimates by the outcome standard deviation (SD=0.99). R2 reflects the pseudo r-squared for fixed effects.

**Table S10**

*Demographic Variable Equivalence Across Full Sample and Preregistered Sample*

|  | **Full Sample** | | |  | **Pre-registered Sample** | | |
| --- | --- | --- | --- | --- | --- | --- | --- |
|  | ***M*** | ***F*** | ***p*** |  | ***M*** | ***F*** | ***p*** |
| PsychMD | 0.02 | 0.12 | 0.73 |  | 0.01 | 0.03 | 0.87 |
| cogdevsoc listserv | 0.07 | 0.28 | 0.60 |  | 0.29 | 1.15 | 0.29 |
| Male | 0.13 | 0.63 | 0.43 |  | 0.14 | 0.56 | 0.46 |
| Female | 0.00 | 0.00 | 0.99 |  | 0.04 | 0.16 | 0.69 |
| Non-binary /Transgender /Preferred not to answer | 0.02 | 0.37 | 0.54 |  | 0.03 | 0.79 | 0.38 |
| Age | 3.53 | 0.04 | 0.84 |  | 4.05 | 0.05 | 0.83 |
| PhD Student | 0.35 | 1.55 | 0.22 |  | 0.37 | 1.54 | 0.22 |
| Postdoctoral Researcher | 0.01 | 0.07 | 0.79 |  | 0.04 | 0.18 | 0.67 |
| Faculty | 0.01 | 0.05 | 0.83 |  | 0.17 | 0.73 | 0.40 |
| Masters / Other | 0.00 | 0.00 | 0.98 |  |  |  |  |
| United States | 0.16 | 0.65 | 0.42 |  | 0.04 | 0.19 | 0.66 |
| Canada | 0.13 | 2.01 | 0.16 |  | 0.00 | 0.01 | 0.92 |
| All other countries | 0.03 | 0.20 | 0.65 |  | 0.00 | 0.00 | 0.98 |
| Native English Speaker | 0.06 | 0.25 | 0.62 |  | 0.20 | 0.86 | 0.36 |
| Before Age 6 | 0.00 | 0.03 | 0.86 |  | 0.17 | 2.02 | 0.16 |
| Between Ages 7-10 | 0.02 | 0.17 | 0.68 |  | 0.03 | 0.19 | 0.67 |
| Between Ages 11-14 | 0.13 | 1.68 | 0.20 |  | 0.04 | 0.43 | 0.52 |
| Between Ages 15-18 | 0.01 | 1.82 | 0.18 |  |  |  |  |
| Statistical Courses Taken | 1.02 | 0.14 | 0.71 |  | 0.53 | 0.05 | 0.82 |
| Psychology | 0.01 | 0.10 | 0.76 |  | 0.01 | 0.20 | 0.66 |
| Other Field | 0.39 | 4.73 | 0.03 | * | 0.01 | 0.20 | 0.66 |
| Clinical | 0.20 | 1.97 | 0.16 |  | 0.04 | 2.05 | 0.16 |
| Cognitive | 0.02 | 0.11 | 0.74 |  | 0.31 | 1.47 | 0.23 |
| Neuroscience | 0.00 | 0.01 | 0.92 |  | 0.00 | 0.05 | 0.83 |
| Developmental | 0.05 | 0.22 | 0.64 |  | 0.08 | 0.31 | 0.58 |
| Quantitative | 0.01 | 0.16 | 0.69 |  | 0.13 | 1.13 | 0.29 |
| Social Personality | 0.06 | 0.47 | 0.49 |  | 0.00 | 0.02 | 0.89 |
| Educational | 0.12 | 1.66 | 0.20 |  | 0.20 | 2.06 | 0.16 |
| Experimental | 0.01 | 0.42 | 0.52 |  |  |  |  |
| Forensic | 0.01 | 1.76 | 0.19 |  | 0.02 | 1.29 | 0.26 |
| History | 0.01 | 1.82 | 0.18 |  |  |  |  |
| Industrial | 0.00 | 0.19 | 0.67 |  | 0.00 | 0.06 | 0.80 |
| School | 0.01 | 1.82 | 0.18 |  | 0.04 | 2.31 | 0.13 |
| Survey Duration | 2578029 | 1.75 | 0.19 |  | 3920048 | 1.26 | 0.27 |
| Excluded | 0.65 | 2.73 | 0.10 |  |  |  |  |

*Note.* *** *p*< 0.001, ***p*<0.01, **p*< 0.05. Each estimate was derived from an One way ANOVA in which the demographic variable was regressed on the Condition variable separately for the full sample and for the pre-registered sample. PsychMD = Psychological Methods Discussion Facebook group. *F* is ANOVA *F*-statistic for the condition variable with 4 categories. Descriptive statistics of full sample and restricted pre-registered analytic sample after cases that met exclusionary criteria were removed. Participants that preferred not to answer questions or did not answer were reported together to prevent any possibility of identification, such as by grouping non-binary and transgender groupings as well as countries of employment. Missing rows indicate that individuals with those demographic variables were not present in the sample.
