# Supplementary material for: Hedges, mottes, and baileys: Causally ambiguous statistical language can increase perceived study quality and policy relevance
Source: PLoS One. 2023 Oct 26;18(10):e0286403. doi: 10.1371/journal.pone.0286403 (PMC10602341; doi:10.1371/journal.pone.0286403)
Supplement: Attachment — Submitted filename: Response to Reviewers 221207.docx [file pone.0286403.s002.docx]

PONE-D-22-24784

Hedges, mottes, and baileys: Causally ambiguous statistical language can increase perceived study quality and policy relevance

PLOS ONE

Dear Dr. Jamie Males,

Thank you for giving us the opportunity to submit a revised version of our manuscript PONE-D-22-24784 “Hedges, mottes, and baileys: Causally ambiguous statistical language can increase perceived study quality and policy relevance.” We sincerely appreciate the detailed and helpful feedback you provided.

Below, we describe how we addressed the reviewer’s comments. For the reviewer’s convenience, we have included all the reviewers’ comments, with our replies and relevant quotes from the revised manuscript following in *italics*.

Sincerely,

Daniela Alvarez-Vargas

**Review Comments to the Author**

Reviewer #1: The manuscript investigates how abstracts that describe their findings with causally ambiguous language (versus causal explicit language) are perceived and evaluated by expert readers (psychology faculty, postdocs, and doctoral students). I think this is an interesting and important study because it informs researchers about the consequences of using causally ambiguous language in reports about nonexperimental research. I also liked that they used random intercepts in their data analysis to deal with the fact that responses were nested within participants. I have reviewed a previous version of the manuscript for another journal. I was happy to see that the authors heeded several of the points that I made in my last review. Here are some comments that might help to further improve the manuscript:

(1) I agree with almost everything stated in the Introduction. However, my impression was that the Introduction section was a bit too long, repetitive, and rambling. I feel like the Introduction could and should be streamlined. For example, I would omit information from the Introduction that is not essential for the current research.

*We have omitted information from the introduction section that was not essential and now our introduction extends from page 4 to page 9.*

(2) The following paragraph—which consists of only one sentence—was not very clear to me (especially the part about “focusing more on their preferred causal interpretation than on ruling out plausible alternatives”): “Other common ways in which psychologists may implicitly communicate a preferred causal interpretation while hedging to avoid criticism for making causal claims on the basis of correlational data include 1) focusing more on their preferred causal interpretation than on ruling out plausible alternatives, or 2) designing studies for which results are far more interesting or important if they are causally informative.“ I would remove or revise this paragraph.

*This paragraph has been revised and now reads as follows: In summary, common ways in which psychologists may communicate a preferred causal interpretation while hedging against causal claims is by 1) focusing more on their preferred causal interpretation than on ruling out plausible alternatives, or 2) designing studies for which results are far more interesting or important if they are causally informative. (p. 8)*

(3) Method section (page 11): “encompassing a variety of interdisciplinary subfields, as shown in Table 2.” I think the authors meant Table 1 (not Table 2).

*We have made this correction on page 11.*

(4) Table 2: The two versions of each abstract differ in terms of causal language at three places. Only two of the three differences are bolded in Table 2. I think the third difference (“impact” vs. “predict” and “affect” vs. “predict”, respectively) should be bolded as well.

*We have boldened the words, accordingly, thank you for your attention to this!*

(5) The page number is missing at some pages after page 14.

*We have corrected this mistake.*

(6) Page 23: I think something is wrong with the following sentence: “Participants rated abstracts that used causally ambiguous statistical language as supporting policy conclusions as strongly as more strongly than causally worded abstracts in Model 1 […] .“ Is there a typo in the sentence? Is there an “or” missing between “as strongly” and “as more strongly”? The sentence is also very long—I didn’t quote the entire sentence. I would recommend to revise the original sentence and make two or three sentences out of it. In general, I would try to avoid sentences that go over more than 2-3 lines.

*We have revised the original sentence and the new one on page 22 is as follows: Participants rated abstracts that used causally ambiguous statistical language as supporting policy conclusions as strongly, or more strongly, than causally worded abstracts in Model 1. Estimates in Models 2-4 were similar to Model 1 in magnitude but statistically nonsignificant and ranging from .16 to .25 points on the 1-5 Likert scale. The preregistered Model 4 generated the largest point estimate, but it was not statistically significant, because over half of participants were excluded.*

(7) Page 33: Did the authors actually mean “ratings of policy relevance” in the following sentence? “indeed, exploratory subgroup analyses suggested that ratings of policy relevance were only affected within participants recruited from one of the three sources“
I guess what the authors actually wanted to say is “support for the conclusion in the final sentence.”

*We have made this correction at the bottom of page 32: “The exploratory subgroup analyses suggested that support for the conclusion in the final sentence of the abstract was only significantly affected within participants recruited from one of the three sources (see Table S4).”*

(8) Page 34: The following sentence was too unspecific: “Participants reliably rated abstracts worded in causally ambiguous statistical language as of higher quality than causally worded abstract” Participants did not rate the quality of the abstracts, did they? They rated the quality of the study design and analysis.

*This is correct; thank you for pointing this out. The sentence on at the bottom of page 33 now reads: “Participants reliably rated the quality of the study design and analysis as higher when the abstract was worded in causally ambiguous statistical language than when it was worded in straightforward causal language.”*

(9) Similarly, the following text in the Abstract was too unspecific: “[participants] rated abstracts from hypothetical studies with causally ambiguous statistical language as of higher quality“ They rated the quality of the study design and analysis (not the quality of the abstract).

*We made this change in our abstract.*

(10) Similarly, I found the second sentence of the Abstract in need of revision. Here is the original version of the sentence: “However, this norm may lead to higher ratings of study quality and greater acceptance of policy recommendations that rely on causal interpretations of nonexperimental findings.“ I would be clearer and more accurate to write: “However, causal ambiguous language may inhibit a critical evaluation of the study design and analysis and lead to a greater acceptance of policy recommendations that rely on causal interpretations of nonexperimental findings.

*We have made the sentence change as suggested.*

(11) I found the following sentence on page 35 difficult to understand: “Taken together, the current study found that the causally ambiguous language did not prevent participants from viewing nonexperimental work as supporting policy conclusions that rely on causal evidence, to the same extent as the same work when described with straightforward causal language.” I think it would be good to revise this sentence.

*We have revised this sentence on page 33 and the sentence now reads as follows: Taken together, we find that using causally ambiguous language instead of straightforward causal language did not prevent participants from viewing nonexperimental work as supporting policy conclusions that rely on causal evidence .*

(12) I noticed that the authors quote the preprint of the taboo paper in the References section. The manuscript has been published at a journal in the meantime: see [https://doi.org/10.1177/1745691620921521](https://urldefense.com/v3/__https:/doi.org/10.1177/1745691620921521__;!!CzAuKJ42GuquVTTmVmPViYEvSg!I-g9NixqsFKLC0-wfaZuS5tuXDpRR-uPUN8FsH0jKfjR8sffsbpTHKj8XMvLnuRoHEjYFofimzcfeM0k$)

*Thank you for this citation we have updated our references.*
