# Supplementary material for: Hedges, mottes, and baileys: Causally ambiguous statistical language can increase perceived study quality and policy relevance
Source: PLoS One. 2023 Oct 26;18(10):e0286403. doi: 10.1371/journal.pone.0286403 (PMC10602341; doi:10.1371/journal.pone.0286403)
Supplement: Attachment — Submitted filename: 230120 Response to reviewers.docx [file pone.0286403.s003.docx]

PONE-D-22-24784R1

Hedges, mottes, and baileys: Causally ambiguous statistical language can increase perceived study quality and policy relevance

PLOS ONE

Dear Dr. Diego A. Forero,

Thank you for giving us the opportunity to submit a revised version of our manuscript PONE-D-22-24784R2 “*Hedges, mottes, and baileys: Causally ambiguous statistical language can increase perceived study quality and policy relevance*.” We sincerely appreciate the detailed and helpful feedback you provided.

Below, we describe how we addressed each of the reviewers’ comments. For the reviewers’ convenience, we have included all the reviewers’ comments, with our replies and relevant quotes from the revised manuscript following in blue *italics*.

Sincerely,

Daniela Alvarez-Vargas

Reviewer #1: I would like to thank the authors for addressing the points that I raised. I was satisfied with how they addressed all my points except for how they addressed point #2.

Here is the original point #2:

“(2) The following paragraph—which consists of only one sentence—was not very clear to me (especially the part about “focusing more on their preferred causal interpretation than on ruling out plausible alternatives”): “Other common ways in which psychologists may implicitly communicate a preferred causal interpretation while hedging to avoid criticism for making causal claims on the basis of correlational data include 1) focusing more on their preferred causal interpretation than on ruling out plausible alternatives, or 2) designing studies for which results are far more interesting or important if they are causally informative.“ I would remove or revise this paragraph.”

Here is how the authors responded:
“This paragraph has been revised and now reads as follows: In summary, common ways in which psychologists may communicate a preferred causal interpretation while hedging against causal claims is by 1) focusing more on their preferred causal interpretation than on ruling out plausible alternatives, or 2) designing studies for which results are far more interesting or important if they are causally informative. (p. 8)”

I was surprised that the authors wrote “in summary” at the beginning of the revised sentence because the sentence does, in my opinion, not or only partially summarize what the authors stated earlier in the text of the paragraph/Introduction. I do not think that the authors talk about “focusing more on their preferred causal interpretation than on ruling out plausible alternatives” earlier in the paragraph/Introduction. Earlier in the paragraph, the authors talked about ambiguous causal language that allows researchers to entertain an interesting causal interpretation of a finding while it allows them to retreat to a boring but more easily defendable noncausal interpretation.

Did the authors also talk about “designing studies for which results are far more interesting or important if they are causally informative”? Relatedly, it was not completely clear to me (a) what these two “common ways” (1 & 2) refer to exactly. If these two ways are indeed distinct from the ways described earlier in the manuscript, I think it would be good to more clearly describe each of the two ways and perhaps provide an example for each way.

Perhaps it might then be good to devote an entire paragraph to these two additional common ways in which psychologists may communicate a preferred causal interpretation while hedging against causal claims. If the two ways are not distinct from what was said earlier in the manuscript, then I would suggest revising the description of the two ways so that it becomes clear that these two ways are similar to what was said earlier. A third alternative option would be to delete the entire sentence from the manuscript. I think the Introduction would also work without this sentence.

hope that helps,
Michael P. Grosz

*The reviewer’s suggestions here were very helpful. We have edited the sentence and also clarified how the two common ways psychologists communicate causal interpretations while hedging against causal claims were explained in earlier paragraphs. Please find the modified paragraphs shown below with examples showcasing how psychologists use these strategies.*

*“By contrast, a researcher who claims they are interested in which kinds of early skills “predict” later achievement may design a study based on its causal interpretation while only analyzing data in such a way that supports the causal interpretation without ruling out plausible alternatives. For example, instead of selecting covariates based on their hypothesized causal relation to the key predictor and outcome of interest [1,9,10,11,12,13,14] , a researcher might include a more limited set of covariates based on common practice within the field or what the researcher finds most interesting. The researcher might address a skeptical reviewer’s concerns by explaining in the discussion section that no causal conclusions can be drawn from nonexperimental studies although the value of the study lies in its contribution towards understanding causal mechanisms. In addition, the researcher might explain that they chose not to consider alternative models because the study’s goals were predictive and not causal. In this process, the researcher would have failed to rule out plausible alternative models. Consequently, the study might avoid the stringent standards of causal evidence to which it would have been subjected if it had explicitly made causal claims.” (p.6-7)*

*We also explain how authors might use both of these strategies in the second paragraph on* page 8 as shown below:

*Additional ways psychologists may implicitly communicate a preferred causal interpretation while hedging to avoid criticism for making causal claims based on correlational data include 1) designing studies for which results are far more interesting or important if they are causally informative and 2) focusing more on their preferred causal interpretation than on ruling out plausible alternatives. For example, consider a hypothetical study that regresses children’s achievement test scores on previous participation in a program designed to improve parent-child interactions, along with a rich set of baseline measures of child and parent skills and contexts, estimating a relation between program participation and adjusted achievement test scores of 0.10 SD. This finding would not be very useful for predictive purposes, nor would it be a useful way to model the process through which some families came to participate in the program and others did not (because test scores are measured after program participation, and thus may be caused by participation). However, if the findings are interpreted as informing readers about the effects of program participation on achievement test scores, a causal interpretation, they are interesting and policy relevant. Further, such recommendations are often given in ways that imply stronger evidence for the relevant causal pathway than* *warranted by the method (Haber et al., 2022), consistent with the hypothesis that the use of causally ambiguous statistical language may be an example of “strategic ambiguity” (Frankenhuis, Panchanathan, & Smaldino, 2022; Rohrer, 2021). Moreover, the method used may focus more on the author’s preferred causal interpretation than on ruling out plausible alternatives, for example by failing to probe the robustness of results to the inclusion of child prior test scores in the model. For these reasons, avoiding the use of causal language completely is unlikely to prevent readers from treating results as if they are intended to be interpreted as causally informative (because of point 1 above), yet the most plausible set of causal estimates will frequently go unreported (because of point 2). (p.8-9)*

Reviewer #2: This manuscript provides an interesting consideration of causal wording and the implications for a readers’ interpretation of the study quality. The study is well designed, and the analyses are appropriate. My main concerns are the proportion of participants who did not pass the manipulation question and the use of “predict” as the non-causal word choice in the sample abstracts.

I found the discussion of predictive models in the introduction section a bit inconsistent and therefore confusing. As the authors’ note in the introduction (no line numbers were included in manuscript), if the purpose of the model is explicitly predictive, then the objective is not to imply an underlying causal mechanism. With this, I agree. However, in several places in the introduction, it is implied that predictive models should be included in a discussion causal wording, presumably because those producing predictive models might use them for intervention purposes? Perhaps this goes back to a need for authors of non-experimental studies to be explicit as to the objective of their study.

If it is to predict an outcome or to (just) identify associations for further study, then causation is not an aim and it is not necessary to control for confounding (although, the misuse of causal language may still be an issue for these studies). Based on this, I think it is unfortunate that the authors’ chose to use “predict” as their non-causal word in the abstracts evaluated by participants. Is it possible that some of the differences in perceived quality are based on the reader’s understanding of the study purpose (predictive versus causal inference), rather that the causal versus non-causal wording?

*The reviewer raises two interesting points. First, studies that have the primary purpose of prediction for prediction’s sake might not need to discuss their causal assumptions or provide a straightforward causal interpretation of their key estimates. We agree with this point, as stated in the introduction. However, in much psychological research and in the abstracts we used as stimuli, the data are analyzed as if the authors are making some effort to obtain a causal estimate (e.g., by statistically controlling for plausible confounders), results are discussed as if they are causally informative, and no use cases for statistical prediction are given (e.g., a range of scores that could be used as a diagnostic cutoff for some useful clinical purpose). In these cases, we think the author and readers are likely to be interested in causal estimates and discussing assumptions and interpretations of the key coefficients are good practices.*

*The reviewer also mentions the possibility that a reason many participants failed the manipulation check is that some viewed the study with “predictive” wording as causal and failed the manipulation check whereas others viewed the study as predictive for prediction’s sake and did not. On one hand, we do attribute the poor performance on the manipulation check to the causal ambiguity of the term “predict”. On the other hand, as can be seen from comparing the estimates from Models 3 and 4 in Table 5, readers who passed the manipulation check were slightly MORE likely to endorse the statement that the predictive study supported the policy conclusions warranting causal evidence. Thus, the idea that these participants were merely viewing the predictive study as not causally relevant seems unlikely.*

Sentence at end of page 4 starting with “Overall, impeding progress ….” Is an incomplete sentence. In the next sentence, “though” should be “although”.

*Thank you for this we have changed the sentence at the end of page 4 to say :*

*“Although disregarding the causal informativeness of study’s design and analysis can reflect a lack of training in causal inference methods, it may also perpetuate a status quo in which the norm of using causally ambiguous statistical language precludes progress toward more causally informative research in nonexperimental psychology.” (p.4-5)*

To me, one of the more interesting findings of this manuscript was the proportion of individuals who erred on the manipulation question was approximately half. This was only superficially discussed but could relate to a general lack of understanding of what words have causal implications. It also may relate to my previous comment on the choice to use “predicts” as the non-causal wording in the abstracts, rather than “associated with” or some other phrase that isn’t associated with a different type of study objective (like predictive modelling). The authors did consider the implications by conducting different model specifications using (as one model) only participants passing this question. Nonetheless, the issue of word choice and the question of why so many participants did not pass the manipulation question could be more comprehensively discussed.

*We completely agree and have included these points in the limitations section of the manuscript as shown below:*

*“Second, we only tested two different types of abstracts with the term “predict” reflecting the causally ambiguous condition. A potential issue with using the term “predict” is that individuals may have interpreted it as reflecting a study exclusively concerned with predictive modelling. This likely explains the high error rate on our manipulation check. Future work might benefit from using words such as “associate” and “link” which Haber and colleagues [17] identified as being more common and causally ambiguous than the term “predict” – although at least half of reviewers rated all three of these words as suggesting a causal link. Whether this would improve nonexperimental research on net would depend on the extent to which these words increase perceived study quality yet continue to convey some degree of causal information to the reader. Finally, perhaps most important for guiding best practices related to causal language is the extent to which wording affects the author’s thinking about causal assumptions and interpretations, which we hope future work will measure.” (p.32-33)*
