# Supplementary material for: Hedges, mottes, and baileys: Causally ambiguous statistical language can increase perceived study quality and policy relevance
Source: PLoS One. 2023 Oct 26;18(10):e0286403. doi: 10.1371/journal.pone.0286403 (PMC10602341; doi:10.1371/journal.pone.0286403)
Supplement: Attachment — Submitted filename: PONE-D-22-24784_R2_review.docx [file pone.0286403.s004.docx]

Dear editor,

Thank you for sending the paper titled, ‘Hedges, mottes, and baileys: Causally ambiguous statistical language can increase perceived study quality and policy relevance,’ for me to review. In this paper, the authors provide an overview of the use of causal language in observational studies, focusing on the examples from psychology. They also conduct a survey based study to assess how causal vs. more statistical language influences readers’ interpretation of study results and find that more ambiguous statistical language leads readers to rank papers as higher quality. The authors appropriately note that the common practice of using ambiguous language may be intended as a cautionary step by authors to avoid over interpretation of results. However, in cases in which explicit hypotheses are being tested, the use of non-causal language may instead lead both authors and readers to overlook important assumptions and sources of bias, without imparting a more cautious interpretation of the results or policy recommendations. Overall, I found the paper topic to be both important and timely. Moreover, I agree with previous reviewer’s comments and was glad to see that the authors addressed these issues. In addition to these broad sentiments, below I pose a few questions and provide some (minor) suggestions that I hope improve the manuscript. Finally, I note that I am not a psychologist, so I refrained from commenting on discipline specific content, e.g., how psychology research informs public policy.

**Questions:**

1. In the introduction section, ‘Potential costs and benefits of a norm against causal language,’ the authors present the argument that despite research that is often based on testing hypotheses, authors often avoid the use of causal methods and causal language, while still making interpretations and recommendations in the discussion of their papers that are based on causal relationships. I thought the authors provide an even handed discussion here, and I agree that a mismatch between the study question, the methods, the language in how results are presented, and the interpretation of research/policy recommendation is problematic. This section could, in my opinion, be even more direct in emphasizing the importance of clear study question that matches the analytical approach. The author’s might recommend that papers identify the data analysis task upfront (i.e., description, prediction, association, or causal inference), as a way to guide the analysis and reduce ambiguity in the interpretation of results (Cf. <https://doi.org/10.1080/09332480.2019.1579578>; <https://doi.org/10.1098/rspb.2020.2815>; <https://doi.org/10.1002/ecy.3336>). In summary, I think the introduction would be more impactful if the authors explicitly identify and create examples based on a specific data analysis task. From here, they can describe how results can be confused by ambiguous language that does not reflect the study question/data analysis task (e.g., a causal model is built but causal language and key assumptions are excluded from the text, or a predictive model is built and causal implications are made in the text). Currently, it is not always clear what data analysis task applies to the example, which makes it harder for the reader to know how they *should* interpret the results.
2. On a related note, I agree with the previous reviewer and the authors that the use of the word prediction/predictor should be used for studies with the explicit goal of prediction. Therefore, depending on the context, I would use a more general word to describe x variables (e.g., explanatory variable, independent variable etc.).
3. Discussing different types of bias that can be introduced when failing to adjust or over adjusting in models used for causal inference might further help clarify to the reader why language should reflect the study question and analytical methods.
4. I think empirical part of this study uses a clever experimental design. My apologies if I overlooked this, but were the treatments random with respect to the background characteristics…both in the total sample (n=142) and the reduced sample (n=55)?
5. This is merely a note, but I found the discussion and the warnings/recommendations therein to be well written and something every-reviewer/author should consider…i.e., the use of causal language, when assumptions are explicit and sources of bias recognized, is much more useful than sweeping those details under the veil of statistically ambiguous language and proceeding boldly with the inference/policy recommendations.

**Minor Comments:**

1. Pg 5, last paragraph. A fewer number of variables and the use of a single model does not distinguish a predictive model from a causal model (e.g., predictive models often perform model selection to identify the set of variables that explain the most variation in an outcome…this process often involves multiple models and possibly many variables since a predictive model is not concerned about relationships among the independent variables). Related to my first comment in the ‘questions’ section, I wonder if clarifying the examples and then highlighting how they can be mis-interpreted causally, would be helpful.
2. Pg 7, first paragraph. I would disagree that causal inference is ‘much more attractive’ than prediction. They are simply different analytical tasks to answer different questions.
3. Pgs 8-9. This exact sentence is repeated on these pages, “such recommendations are often given in ways that imply stronger evidence for the relevant causal pathway than warranted by the method [17] consistent with the hypothesis that the use of causally ambiguous statistical language may be an example of “strategic ambiguity” [20, 22].”
4. Pg 9, last sentence. Why does a more complicated model result in a smaller estimate?
5. Pg 10. What does the effect size of 0.30 represent…a difference in some unitless score?
6. Pgs 12-13. The responses to the number of individuals who failed the manipulation check are contradictory…is it 79, as noted on pg 12, or 64 as indicated on pg 13. In both cases, these are not 44% of 142.
7. Pg 13 and 18. Final sample sizes after exclusion criteria (n=55, pg 13) and in the final model (n=58, pg 18) do not agree.
8. Table 2, column headers are not consistent…Abstract 1 does not include sample size in header like Abstract 2.
9. In Tables 4 and 5, the subtitle says that model 1 uses only the first abstracts read (“Model 1 is an ordinary least squares model using only the quality response from the first abstract seen by the participants.”), but in fact based on the model specifications table and the table above that including the estimates, this must be incorrect (e.g., model 2 has no estimate for order).
10. Figure 1 caption: subject verb agreement issue, “abstracts that *have”*
