# Supplementary material for: Hedges, mottes, and baileys: Causally ambiguous statistical language can increase perceived study quality and policy relevance
Source: PLoS One. 2023 Oct 26;18(10):e0286403. doi: 10.1371/journal.pone.0286403 (PMC10602341; doi:10.1371/journal.pone.0286403)
Supplement: Attachment — Submitted filename: Response to Reviewers Letter 23 05 08.docx [file pone.0286403.s005.docx]

PONE-D-22-24784R1

Hedges, mottes, and baileys: Causally ambiguous statistical language can increase perceived study quality and policy relevance

PLOS ONE

Dear Dr. Diego A. Forero,

Thank you and the reviewers for giving us the opportunity to submit a revised version of our manuscript PONE-D-22-24784R2 “*Hedges, mottes, and baileys: Causally ambiguous statistical language can increase perceived study quality and policy relevance*.” We have addressed each of the reviewers’ comments. For the reviewers’ convenience, we have included all the reviewers’ comments, with our replies and relevant quotes from the revised manuscript following in blue *italics*.

Sincerely,

Daniela Alvarez-Vargas

**Reviewer #3:**

Dear editor,
I have read through the authors' point-by-point response and the revisions that they made to the manuscript titled, ‘Hedges, mottes, and baileys: Causally ambiguous statistical language can increase perceived study quality and policy relevance’. Upon reading through point-by-point, I found that the authors did a nice job of addressing my prior suggestions. Here, I provide a few final suggestions for their consideration.

1. In the introduction, starting on page 5, the first sentence the authors added in the revised draft seems a bit unclear to me. When they state, “...analytic approach does not match the interpretation of the implications of the results.” I am no sure what is meant by implications...maybe policy implications, or maybe the word implications can be omitted altogether.

*We have revised this sentence to clarify that we are discussing the policy implications. The first sentence on page 5 now reads:*

*A possible unintended consequence of the norm, however, can occur when the authors research questions and analytic approaches do not match the interpretation of the policy implications of the results.*

2. A few lines down on from the previously mentioned sentence on page five, ‘two variables are discussed’ in the context of a simple regression model. More specifically, they state “...upwardly bias the estimated effect of children’s early academic skills if instead of selecting covariates based on their hypothesized causal relation to the key explanatory variable and independent variable of interest...” I think the authors might mean dependent variable of interest, since confounding variables are both associated with the explanatory variable and the outcome.

*We have corrected this sentence and it now reads as follows: This analytic approach would upwardly bias the estimated effect of children’s early academic skills if instead of selecting covariates based on their hypothesized causal relation to the key explanatory variable and dependent variable of interest [1,4,5,6,7,8,9], a researcher includes a more limited set of covariates based on common practice within the field.*

3. Regarding question 4 from my previous review, the authors asked if they needed to reword their revision...I have no problem with them incorporating text from my review into their manuscript.

*Thank you for your this!*

4. In my previous minor revision #4, the authors response in which they note that adjustment for confounds may lead to a smaller estimate of interest, they then say that “...a straightforward causal framing of the reduced estimates may be detrimental...” I think this sentence could be clearer. The reduced size of the estimate may lead some policy makers to perceive the study as less conclusive, but the explanation of causal inference methods per se should speak to the robustness of the relationship between x and y, since causal inference can help explain why estimates change from one model to the next.

*This is a great point for clarification; we have modified the sentence on page 10 as shown below:*

*In addition, contrasting estimates from increasingly well-controlled models to account for confounding variables or conduct sensitivity and robustness tests often yields smaller associations between the key dependent and independent variables. When this is the case, a straightforward causal framing of the reduced estimates speaks to the robustness of the relationship between the key dependent and independent variables. However, it may be detrimental for the perceived quality and policy-relevance of one’s work if policy makers perceive the study to be less conclusive.*
